# The Immunomodulatory Effects of Honey and Associated Flavonoids in Cancer

**DOI:** 10.3390/nu13041269

**Published:** 2021-04-13

**Authors:** Razan J. Masad, Shoja M. Haneefa, Yassir A. Mohamed, Ashraf Al-Sbiei, Ghada Bashir, Maria J. Fernandez-Cabezudo, Basel K. al-Ramadi

**Affiliations:** 1 Department of Medical Microbiology and Immunology, College of Medicine and Health Sciences, United Arab Emirates University, Al Ain, United Arab Emirates; 201890026@uaeu.ac.ae (R.J.M.); shojamh@uaeu.ac.ae (S.M.H.); yassirmohamed@uaeu.ac.ae (Y.A.M.); ghadab@uaeu.ac.ae (G.B.); 2 Department of Biochemistry and Molecular Biology, College of Medicine and Health Sciences, United Arab Emirates University, Al Ain, United Arab Emirates; 201180866@uaeu.ac.ae (A.A.-S.); mariac@uaeu.ac.ae (M.J.F.-C.); 3 Zayed Center for Health Sciences, United Arab Emirates University, Al Ain, United Arab Emirates

**Keywords:** honey, flavonoids, cancer, tumor immunomodulation, inflammation, complementary medicine

## Abstract

Honey has exerted a high impact in the field of alternative medicine over many centuries. In addition to its wound healing, anti-microbial and antioxidant properties, several lines of evidence have highlighted the efficiency of honey and associated bioactive constituents as anti-tumor agents against a range of cancer types. Mechanistically, honey was shown to inhibit cancer cell growth through its pro-apoptotic, anti-proliferative and anti-metastatic effects. However, the potential of honey to regulate anti-tumor immune responses is relatively unexplored. A small number of in vitro and in vivo studies have demonstrated the ability of honey to modulate the immune system by inducing immunostimulatory as well as anti-inflammatory effects. In the present review, we summarize the findings from different studies that aimed to investigate the immunomodulatory properties of honey and its flavonoid components in relation to cancer. While these studies provide promising data, additional research is needed to further elucidate the immunomodulatory properties of honey, and to enable its utilization as an adjuvant therapy in cancer.

## 1. Introduction

Cancer represents a crucial health burden, and is considered as the second leading cause of death worldwide, accounting for 10 million deaths annually [1]. According to the International Agency for Research on Cancer, 19.3 million new cases were estimated in 2020 [1]. Cancer is a multistep process that starts from a single transformed cell. The pathogenesis of cancer is characterized by a shift in cellular proliferation, invasion, and metastasis capacities. This process is controlled by various transcription factors, protein kinases, cell cycle proteins, pro-apoptotic and anti-apoptotic proteins, and other molecular targets [2].

Despite the efficiency of currently used anti-cancer drugs, drug-induced toxicity remains a major concern in treatment [3]. Thus, the focus on using alternative natural products for cancer prevention and treatment has been increasing over the past few years. Among these natural products, honey has been extensively researched.

**Figure 1:**
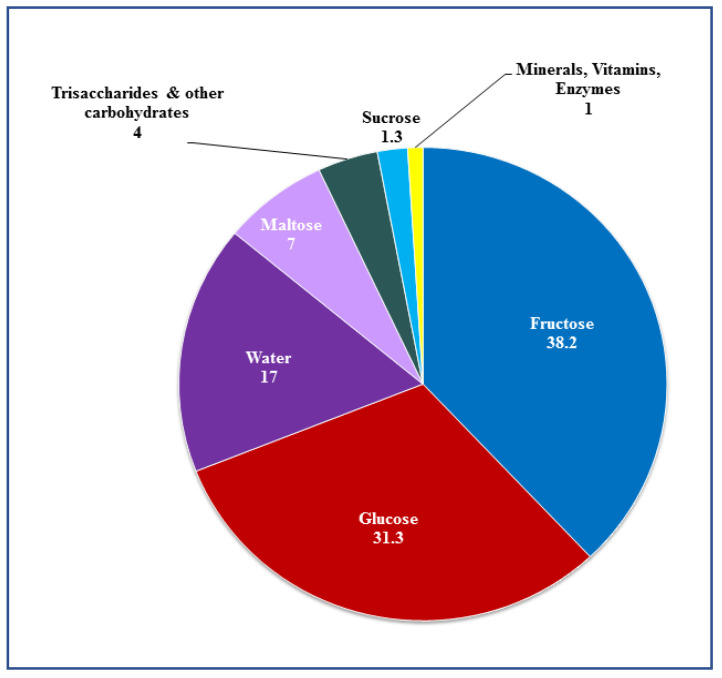
Major constituents of honey.

Over the last decades, honey has been considered as an effective natural medicine for a variety of disorders. The composition of honey is dependent on its floral source as well as its geographical origin. In general, natural honey consists of 75% monosaccharide sugars (31% glucose and 38% fructose), 10–15% disaccharide sugars (mainly sucrose and maltose), as well as a mixture of enzymes, minerals, vitamins, amino acids, flavonoids and phenolic compounds which accounts for the remaining percentage [4] (Figure 1, Table 1). Several studies have shown that honey has antioxidant [5,6,7,8,9], anti-microbial [10], anti-inflammatory [11,12,13], and anti-tumor effects [4,8,14,15,16,17,18,19,20].

The anti-tumor effects of honey have been examined using various cancer cell lines and tissues. In fact, honey was shown to decrease the tumorigenicity of different cancer types, including colorectal [8,15,16,21], breast [14,16,17,18,19,22], lung [19,21,23], skin [16], prostate [20,22,24], renal [25] and cervical cancer [26]. Moreover, it was shown that honey can enhance the effect of chemotherapeutic drugs such as 5-fluorouracil and paclitaxel [16,23]. The anti-tumor effects of honey have been attributed to its role in the induction of apoptosis [16,18,24,27,28], modulation of oxidative stress [5,6,8,9,18,29], and anti-proliferative [8,16,17,18,30,31,32] and anti-metastatic properties [8,9,18,23,30]. In addition, several studies have demonstrated that honey has the ability to modulate the immune system by inducing an anti-inflammatory effect against cancer [8,15,25,33]. The focus of the present review will be the immunomodulatory properties of honey and its constituent flavonoid compounds.

## 2. Immunomodulatory Properties of Honey: Induction of Proinflammatory Cytokines by Myeloid Cells

**Figure 2:**
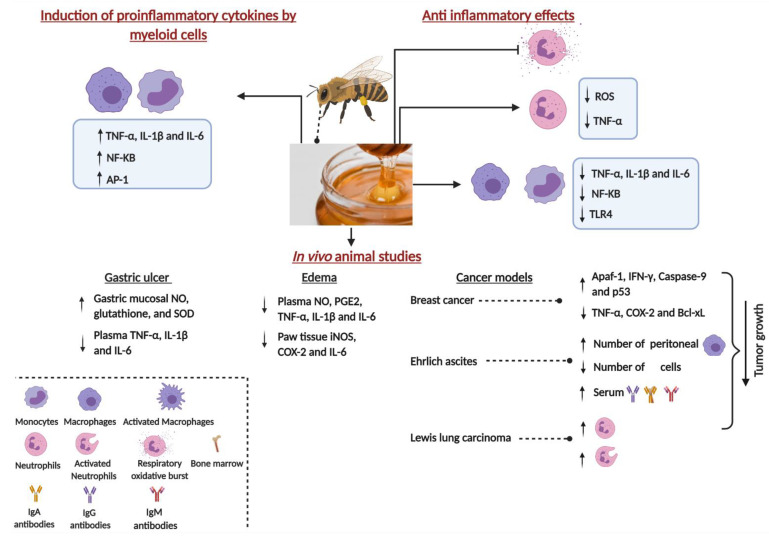
Schematic diagram of the major immunomodulatory effects of honey. Created with BioRender.com. TNF-α, Tumor Necrosis Factor-alpha; IL-1β, Interleukin 1-beta; IL-6, Interleukin-6; NF-kB, Nuclear factor kappa B; AP-1, Activator Protein - 1; NO, Nitric Oxide; SOD, Superoxide Dismutase, IL-6, Interleukin 6, PGE2, prostaglandin E2; iNOS, Inducible Nitric Oxide Synthase; COX-2, Cyclooxygenase-2; ROS, Reactive Oxygen Species; TLR4, Toll-Like Receptor 4; Apaf-1, Apoptotic Protease Activating Factor 1, IFN-γ, Interferon gamma; p53, Cellular Tumor Antigen p53; Bcl-xL, B-Cell Lymphoma-Extra Large; IgA, Immunoglobulin A; IgG, Immunoglobulin G; IgM, Immunoglobulin M.

Several lines of evidence have highlighted the efficiency of honey as an immunomodulatory agent (Figure 2). In a study published nearly 20 years ago, exposure of the MM6 monocytic cell line to manuka and pasture honeys led to a significant reduction in the release of the reactive oxygen species (ROS). This reduction was accompanied by a significant enhancement in the production of the proinflammatory cytokine tumor necrosis factor-alpha (TNF-α). The mechanism by which honey induces its immunomodulatory effects was suggested to be due to the increased levels of hydrogen peroxide in the honey that may have resulted in a negative feedback effect on the release of ROS by MM6 cells [34].

Consistent with these results, a significant increase in the production of TNF-α, interleukin-1 beta (IL-1β) and IL-6 cytokines was reported following incubation of MM6 cells or human blood monocytes with jelly bush, manuka or pasture honey [35]. In this study, jelly bush honey was found to result in a significantly higher cytokine release compared to the other honey types, but the underlying reason for this activity is not known. Following fractionation of manuka honey, a 5.8 kDa, heat-sensitive component was identified to be responsible for the induction of cytokine release from MM6 cells, murine bone marrow derived macrophages (BMDM) and human monocytes [36]. This induction was reported to be driven via interaction with Toll-like receptor 4 (TLR4) on immune cells, as the blocking of this receptor resulted in an inhibition of the observed honey-mediated immunomodulatory effects. In addition, unlike the response of BMDM isolated from TLR2 knockout mice, BMDM isolated from TLR4 knockout mice were stunted in their cytokine production after stimulation with the 5.8 kDa component of manuka honey [36].

The endotoxin concentrations in the various honey types used in the above studies were reported to be very low (<1 ng/mL). Honey-mediated induction of cytokines from MM6 cells was significantly reduced by prior heat treatment of honey samples, suggesting that the active component responsible for the induction is heat-sensitive. Moreover, addition of polymyxin B (PMB) to manuka honey (MH)-treated MM6 cells did not affect their cytokine release [35,36]. Given that lipopolysaccharide (LPS) is a heat-stable compound that is inhibited by PMB, the authors proposed that the immunomodulatory effect of honey is independent of its endotoxin concentration. Similarly, thyme honey was also shown to stimulate the activation of nuclear factor kappa B (NFκB) and activator protein-1 (AP-1) transcription factors in RAW 264.7 murine macrophages, leading to the production of prostaglandin E2 (PGE2), TNF-α and IL-6 [37]. Although this stimulation was apparently independent of LPS, the bioactive entity responsible for this activity was not identified.

Another study compared the immunomodulatory effects of New Zealand kanuka, manuka and clover honeys. All three types of honey could stimulate TNF-α release from the human THP-1 monocytic cell line and U937 histiocytic lymphoma, with kanuka honey being the most efficient inducer [38]. The LPS concentrations in the examined honey types were reported to be insufficient to explain their immunostimulatory effect. Instead, the authors identified type II arabinogalactan proteins, members of a large and diverse family of cell surface-expressed proteoglycans present in plant cell walls [39], as the immunostimulatory moiety in kanuka honey [38]. This is an interesting observation since these proteins have been demonstrated to possess immunostimulatory properties in a limited number of studies [40,41]. However, the precise mechanism by which these glycoproteins affect immune cell function remains unknown. These data suggest that honey and its constituents can induce the production of proinflammatory cytokines from myeloid cells. In the context of cancer, such cytokines act to reduce tumor cell growth through their anti-proliferative and pro-apoptotic activities, as well as promoting anti-tumor immune responses [42,43].

In contrast to these findings, other groups favored a role for endotoxin in honey-mediated induction of immune cells. In one study, the authors investigated the effect of Manuka honey, Danish honey and artificial honey on the production of IL-6 by MM6 cells [44]. In agreement with previous studies, both types of honey induced a significant release of IL-6 following overnight incubation. Since it was found that the substance responsible for this activity is heat stable and prone to inhibition by PMB, the authors suggested that the immunostimulatory effects of honey are mainly due to its LPS content. This conclusion was rationalized by the fact that previous studies have reported that very low concentrations of LPS (3.1 pg/mL) were enough to stimulate IL-6 release from MM6 cells [45]. Nevertheless, no attempt to directly identify the active moiety was made in that study. Taken together, the aforementioned studies suggest that some components of natural honey may have direct stimulatory activities on myeloid cells. The contribution of LPS, which is present at variable but generally low concentrations in all natural honey types, to the induction of proinflammatory cytokines in macrophages is still a matter of debate among different researchers in the field. Resolving this outstanding issue will require the use of more genetically refined systems in which, for example, specific components of the TLR signaling pathway are defective.

## 3. Anti-Inflammatory Properties of Honey

In contrast to the above findings, other studies have highlighted an anti-inflammatory role for honey on immune cells. Thrombin-induced oxidative respiratory burst in human neutrophils and rodent peritoneal macrophages was inhibited by co-incubation with different kinds of commercial honey [46]. Likewise, another study indicated a significant, dose-dependent reduction of human neutrophil superoxide production after treatment with three types of New Zealand honeys: rewarewa, manuka and kanuka [46]. In line with these findings, pasture and manuka honeys were shown to have excellent anti-oxidant potential, with the latter being able to effectively quench free radicals within a few minutes [47]. In fact, these quenching properties of MH were attributed to the presence of methyl syringate (MSYR) [48]. Additionally, at a concentration of 400 µg/mL, MH could effectively inhibit the production of TNF-α by neutrophils [49]. However, encapsulation of MH with alpha-cyclodextrin molecules was shown to decrease this activity. This indicates that processing MH into cyclodextrin-based complexes in the form of free-flowing powder reduces the anti-inflammatory capacity of MH [49].

The mechanism(s) by which MH exerts its anti-inflammatory property was recently investigated. MH treatment of LPS-induced RAW 264.7 macrophages mitigated against cellular toxicity and improved cell viability. This effect was correlated with decreased ROS and nitrite accumulation and inhibition of cellular apoptosis [50]. In addition, MH treatment inhibited the TLR4/NF-κB signaling pathway and led to a reduction in the secretion of several proinflammatory cytokines, including TNF-α, IL-1β and IL-6 [50]. Similar findings were also recently reported for Brazilian stingless bee honey [51].

Chronic inflammation has been shown to be linked to cancer progression, as it prevents the healing of the damaged tissues. Various proinflammatory enzymes and cytokines induce the inflammatory process. The cyclooxygenase-2 (COX-2) enzyme is involved in the process of carcinogenesis, where it was found to be overexpressed in several malignant conditions. COX-2 catalyzes the metabolism of arachidonic acid to prostaglandin [52,53], an integral reaction of carcinogenesis and inflammation [54]. The anti-inflammatory activity of honey was attributed to its phenolic compounds and flavonoids. These compounds were demonstrated in several studies to suppress the activity of COX-2 and/or inducible nitric oxide synthase (iNOS), hence resulting in an anti-inflammatory response [53,55,56]. Similarly, flavonoid and phenolic acid extracts of Malaysian honey were shown to have anti-inflammatory and cytoprotective properties [24]. In this study, honey extracts inhibited the release of NO from activated RAW264.7 macrophages and protected cells against TNFα-induced cytotoxicity. The authors suggested that the observed cytoprotective effect could be due to either the free radical scavenging capacity of honey flavonoids [57], or to the induction of cytoprotective enzymes, such as heme oxygenase-1 (HO-1), by flavonoids [58]. Thus, it is clear that honey-related constituents may have pro- or anti-inflammatory properties dependent on the concentrations as well as the target cells and culture conditions used in the various assays.

## 4. Immunomodulatory Properties of Honey: In Vivo Animal Model Studies

Several studies were conducted to investigate the immunomodulatory effect of honey in different disease models. In gastric ulcer-bearing rats, manuka honey treatment resulted in a significant increase in the gastric mucosal levels of nitric oxide (NO), glutathione (GSH) and superoxide dismutase (SOD), in addition to a decline in the plasma levels of TNF-α, IL-1β, and IL-6 pro-inflammatory cytokines [59]. Using an inflammatory model of carrageenan-induced edema, pretreatment with oral Gelam honey could effectively reduce edema in a dose-dependent fashion in inflamed rat paws, decrease the production of NO, PGE2, TNF-α, and IL-6 in plasma, and suppress the expression of iNOS, COX-2, TNF-α, and IL-6 in paw tissue [55].

Other studies have examined the modulatory effects of honey using pre-clinical cancer models. In a carcinogen-induced breast tumor model in Sprague Dawley rats, oral administration of relatively high concentrations (1.0 g/kg body weight per day) of Malaysian Tualang honey (TH) or MH after tumors became palpable was shown to retard cancer growth, resulting in smaller tumor weights and volumes and a better histological grade compared to the untreated group [60]. This was accompanied by an increase in the expression of Apaf-1, Caspase-9 and IFN-γ pro-apoptotic proteins, and a decrease in the expression of TNF-α, COX-2, and Bcl-xL anti-apoptotic proteins [60]. Similarly, another study utilized per oral administration of large daily doses of Egyptian bee honey (at 0.1–10 g/kg body weight) before intraperitoneal inoculation with Ehrlich ascites tumor and demonstrated an inhibition in tumor growth and a decrease in tumor-associated toxicities [60]. The honey treatment resulted in an increase in the number of bone marrow cells and peritoneal macrophages, with a significant enhancement in their phagocytic function. Similarly, treatment of Ehrlich ascites carcinoma-bearing mice with coriander honey (0.5 g/kg body weight per day) resulted in a decrease in the tumor volume, with an augmentation of the levels of serum IgM, IgG and IgA as well as macrophage phagocytic activity [60]. Finally, repeated intraperitoneal administrations of jungle honey (JH) were shown to induce recruitment and activation of neutrophils in C57BL/6 mice. When JH-treated mice were subsequently implanted with Lewis Lung Carcinoma/2 (LL/2) cells intraperitoneally, tumor growth and incidence were significantly inhibited compared to control mice [19]. Thus, intraperitoneal administration of JH appeared to recruit and activate neutrophils which were then able to limit cancer growth within that site. Nevertheless, no direct demonstration of increased anti-tumor activity by peritoneal neutrophils was presented.

## 5. Immunomodulatory Effects of Major Polyphenolic Compounds in Honey

Polyphenols are chemical compounds that arise from secondary plant metabolism and express multiple phenolic groups associated with complex structures [61]. These compounds play critical roles in plant reproduction and growth, as well as protection against UV radiation, mechanical damage and microbial infections [62]. Based on their chemical structure, polyphenols can be divided into flavonoid and non-flavonoid (phenolic acids) compounds. Flavonoids are water-soluble chemical compounds with low molecular weight. They are formed by two benzene rings linked with three atoms of carbon, and a minimum of two phenolic groups (OH). Flavonoids are divided into flavanols, flavones, flavanonols, flavonols, flavanones, isoflavones, anthocyanins and anthocyanidins [63]. Phenolic acids (phenolcarboxylic acids) acquire a phenolic ring and at least one organic carboxylic acid. According to their structure, they can be divided into coumaric, ferulic, caffeic acids, acetophenones, phenylacetic, syringic, vanillic and gallic acids [64]. The phenolic composition of honey depends on its floral source and geographical origin. Table 1 shows the most common phenolic compounds present in different types of honey [24,65,66,67,68,69,70,71,72,73,74,75,76,77,78].

**Table 1:** Major polyphenols in honey.

Type of Honey	Major Flavonoids	Major Phenolic Acids	References
Manuka Honey	Chrysin, Galangin, Isorhamnetin, Kaempferol, Luteolin, Apigenin, Pinobanksin, Pinocembrin, Quercetin	2-methoxybenzoic, 2-methoxybenzoic acid, 2-methoxybenzoic acid, Caffeic acid, Ferulic acid, Gallic acid, p-Coumaric acid, Syringic acid, 2-methoxybenzoic acid	[65,66,70]
Kanuka Honey	Chrysin, Galangin, Isorhamnetin, Kaempferol, Luteolin, Pinobanksin, Pinocembrin, Quercetin	4-methoxyphenyllactic acid, Gallic acid, Abscisic acid, Phenyllactic acid, Syringic acid, Ferulic acid	[66]
Tualang Honey	Apigenin, Catechin, Chrysin, Kaempferol, Luteolin	2-Hydroxycinnamic acid, Caffeic acid, Cinnamic acid, Gallic acid, p-Coumaric acid, Syringic acid	[67]
Acacia Honey	Apigenin, Chrysin, Galangin, Genistein, Kaempferol, Luteolin, Myricetin, Pinobanksin, Pinocembrin, Quercetin	Caffeic acid, Chlorogenic acid, Ferulic acid, Gallic acid, p-Coumaric acid, Syringic acid, Vanillic acid	[68,69]
Strawberry Tree Honey	Apigenin, Galangin, Genistein, Kaempferol, Luteolin, Pinobanksin, Pinocembrin, Rutin	Apigenin, Galangin, Genistein, Kaempferol, Luteolin, Pinobanksin, Pinocembrin, Rutin	[71]
Clover Honey	Quercetin	Cinnamic acid, p-Hydroxybenzoic acid	[66,72]
Heather Honey	Chrysin, Galangin, Myricetin	Chlorogenic acid, Cinnamic acid, Ellagic acid, Ferulic acid, Gallic acid, p-Coumaric acid, p-Hydroxybenzoic acid, Protocatechuic acid, Sinapic acid, Syringic acid, Vanillic acid	[70,73]
Thyme Honey	Chrysin, Kaempferol, Luteolin, Myricetin, Quercetin	Caffeic acid, Chlorogenic acid, Cinnamic acid, Ferulic acid, Gallic acid, p-Coumaric acid, Syringic acid	[70,71,73]
Rosemary Honey	Chrysin, Kaempferol, Luteolin, Pinobanksin, Pinocembrin	p-Coumaric acid	[71,74]
Pine Honey	Quercetin, Genectin, Chrysin, Apigenin, Naringenin, Luteolin, Hesperetin, Rutin	Gallic acid, p-Coumaric acid, Ferulic acid, Caffeic acid, Vanillic acid, Syringic acid	[75]
Gelam Honey	Catechin, Chrysin, Hesperetin, Kaempferide, Kaempferol, Myricetin, Quercetin	Gallic acid, Chlorogenic acid, Caffeic acid, p-Coumaric acid, Ferulic acid, Ellagic acid	[24,76]
Royal Jelly Honey	Apigenin, Chrysin, Fisetin, Galangin, Genistin, Luteolin, Pinocembrin, Quercetin, Naringenin	Ferulic acid, p-Coumaric acid, Caffeic acid	[73,77,78]
Rewarewa Honey	Chrysin, Galangin, Kaempferol, Luteolin, Apigenin, Pinobanksin, Pinocembrin, Quercetin	Gallic acid, Abscisic acid, Phenyllactic acid, Syringic acid, Ferulic acid	[66]

**Figure 3:**
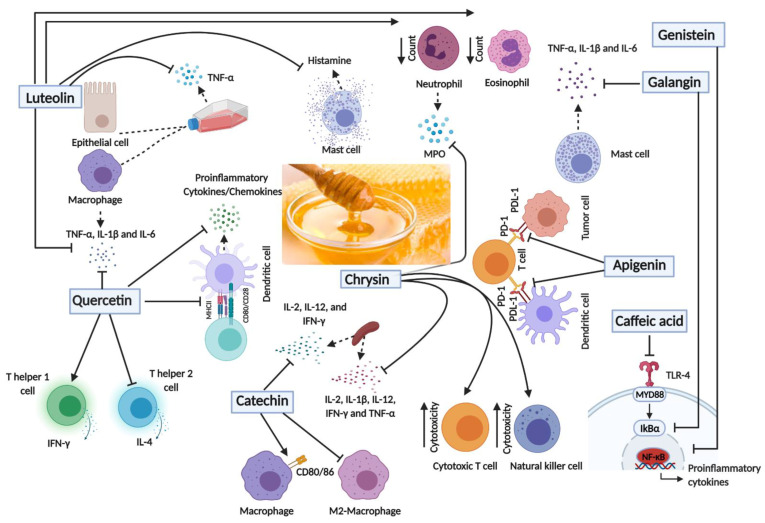
Schematic diagram of the main immunomodulatory effects of the major polyphenols in honey. Created with BioRender.com. TNF-α, Tumor Necrosis Factor-alpha; IL-1β, Interleukin 1-beta; IL-2, Interleukin-2; IL-4, Interleukin-4; IL-6, Interleukin-6; IL-12, Interleukin-12; IFN-γ, Interferon gamma; CD80/86, Cluster of differentiation 80/86; CD28, Cluster of differentiation 28; MHCII, Major histocompatibility complex II; MPO, Myeloperoxidase; PD-1, Programmed Cell Death protein 1,; PDL-1, Programmed Death-Ligand 1; TLR4, Toll-Like Receptor 4, MYD88, Myeloid differentiation primary response 88, IκBα; Nuclear factor of kappa light polypeptide gene enhancer in B-cells inhibitor-alpha; NF-kB, Nuclear factor kappa B.

Several lines of evidence have highlighted the immunomodulatory role of the major phenolic compounds present in honey, sometimes with conflicting findings (Figure 3). In one study, for example, quercetin was found to inhibit LPS-induced release of TNF-α, IL-1β and IL-6 by macrophages by suppressing the activation of the extracellular signal-regulated kinases (ERK) and mitogen-activated protein kinases (MAPK) [79,80]. Similarly, treating murine dendritic cells (DCs) with quercetin resulted in a decrease in the production of proinflammatory cytokines and chemokines. This was accompanied by a decrease in the expression levels of the major histocompatibility complex class two (MHC II) and costimulatory molecules, resulting in an inhibition of T cells activation [81]. The release of IL-6 by human mast cells following Fc epsilon R I (FcεRI) triggering or through nonallergic IL-6-dependent pathways could be effectively inhibited by quercetin [82]. In contrast, however, quercetin treatment of human peripheral blood mononuclear cells was reported to preferentially induce interferon gamma (IFN-γ) expression and synthesis while inhibiting IL-4 production [83]. The data were purported to show that quercetin can differentially activate Th1 cells, thus providing a potential mechanism for the anti-tumor effect of this flavonoid [83].

The immunomodulatory effect of quercetin was also examined in different autoimmune disease conditions. In one study, treating peripheral blood mononuclear cells (PBMCs) isolated from multiple sclerosis patients resulted in a decrease in the proliferation of these cells along with a decrease in the release of TNF-α and IL-1β cytokines [84]. Similarly, quercetin was shown to suppress the secretion of TNF-α and interfere with the onset of disease in an inflammatory bowel disease (IBD) model [85]. In an acute colitis model, quercetin was shown to induce an anti-inflammatory effect that is mediated by an impaired dendritic cell (DC) response [86]. Finally, in experimental autoimmune encephalomyelitis, quercetin blocked Th1 differentiation [87]. Based on available evidence, quercetin appears to possess a wide range of biological activities, including anti-inflammatory, anti-viral, antioxidant and anti-carcinogenic actions [88].

Other polyphenols were also found to have immunomodulatory activities. Luteolin was found to inhibit gene expression of proinflammatory cytokines as well as the release of TNF-α in LPS-induced murine macrophages by reducing activation of ERK and p38 [89,90]. Similarly, luteolin inhibited the secretion of TNF-α and IFNγ-mediated activation of Signal transducer and activator of transcription 1 (STAT1), signal transducer and activator of transcription 3 (STAT3) and cyclooxygenase 2 (COX-2) in murine macrophages [91]. Luteolin also suppressed NFκB activation and TNF-α secretion from cocultured intestinal epithelial cells and RAW 264.7 cells [92]. Moreover, luteolin could inhibit cell proliferation and IFN-γ production of murine and human autoreactive T cells [93]. In disease models, treatment of asthmatic rats with luteolin for 8 weeks resulted in a decrease in the neutrophil and eosinophil counts and suppressed the release of IL-4 in comparison to the control group [94]. In an autoimmune thyroiditis mouse model, luteolin exerted anti-inflammatory effect and inhibited the tyrosine-phosphorylation and activation of STAT3. This treatment resulted in a decrease in lymphocytic infiltration in the thyroid glands and attenuated the destruction of the thyroid follicles [91].

Apigenin treatment was found to decrease LPS-induced luciferase expression in the lungs of NFκB luciferase transgenic mice, which express luciferase under the control of NFκB.This was accompanied by an attenuation of LPS-induced neutrophil infiltration into the lungs, as well as a decrease in the gene expression profile of neutrophil chemotactic factors in the lungs of apigenin treated mice [95]. In a colitis model, apigenin reduced inflammation which was accompanied by a lower score of colonic damage [96,97]. In addition, apigenin treatment normalized the expression of TNF-α, TGF-β and CC chemokine ligand 2 (CCL-2) inflammatory markers [97]. In a murine asthma model, administration of apigenin resulted in an inhibition of ovalbumin (OVA)-induced increase in eosinophils and Th17 cells in sensitized animals [98]. Combining apigenin and luteolin resulted in inhibition of murine and human autoreactive T cell responses [93], suggesting that the anti-inflammatory activities of different flavonoid compounds may be combined to achieve a greater degree of control of unwanted immune responses.

Chrysin was also shown to inhibit the production of proinflammatory cytokines from LPS-induced PBMCs [83]. Oral administration of chrysin reduced the release of IL-6 and IL-1β in cell culture supernatants of colon tissues of IBD-bearing mice [99]. In addition, chrysin treatment resulted in a significant decrease in the myeloperoxidase (MPO) activity in murine neutrophils [99]. Using an experimental autoimmune neuritis (EAN) model, oral administration of chrysin, starting at the onset of symptoms, attenuated the severity and duration of the clinical course of the disease and reduced inflammatory cell infiltration and demyelination of sciatic nerves [100]. This was accompanied by inhibition of the release of IL-2, IL-1β, IL-12, IFN-γ and TNF-α by spleen cells. The efficacy of chrysin as an anti-inflammatory agent has been subsequently demonstrated in other disease models, such as experimental autoimmune uveitis [101], atopic dermatitis [102] and drug-induced pulmonary fibrosis [103].

Genistein, an isoflavone compound, was also able to inhibit LPS-induced activation of NFκB in monocytes [89]. Similarly, galangin was found to halt the degradation of IκBα and translocation of p65 NFκB, leading to a repression in TNF-α, IL-6, IL-1β, and IL-8 gene expression in mast cells [104]. The polyphenol caffeic acid was also shown to suppress the TLR4 pathway and LPS-induced NFκB activation [105]. In contrast, the flavanol compound, catechin, was shown to have immunostimulatory activities, inducing the release of IFN-γ, IL-12 and IL-2 from murine splenocytes, and activating the antigen presentation capacity of macrophages by upregulating the expression CD80 and CD86 costimulatory proteins [106].

The immunomodulatory role of honey polyphenols against cancer was also demonstrated in many studies. For instance, luteolin suppressed the release of the CCL2 chemokine, which is involved in the recruitment of tumor-associated macrophages (TAMs) in the tumor microenvironment. This was accompanied by an inhibition of the migration of Lewis lung carcinoma cells [107]. Apigenin was found to inhibit IFN-γ-mediated STAT1 activation, leading to reduced expression of programmed death-ligand 1 (PD-L1) in A375 melanoma cells, and thus alleviating the PD-1/PD-L1–mediated inhibition of anti-tumor immune responses [108]. Apigenin treatment reduced the expression of PD-L1 in DCs, resulting in enhancement of the host’s T cell immunity [108]. Likewise, quercetin was shown to block the PD-1/PD-L1 interaction [109]. Genistein was found to inhibit M2-polarized macrophages as well as the stemness of SKOV3 and OVCA-3R ovarian cancer cells by disrupting the IL8/STAT3 signaling axis [110]. In a melanoma model, oral treatment of chrysin resulted in suppression of the tumor growth by 60% and 70% following 14 and 21 days of treatment, respectively. This was accompanied by an increase in the killing activity of cytotoxic T cells and NK cells [111]. Likewise, chrysin treatment increased the cytotoxicity of NK cells in isolated splenocytes from leukemic BALB/c mice [112]. Taken together, these findings confirm the effectiveness of various flavonoid compounds in boosting anti-tumor immune responses in preclinical cancer models and suggest that multiple mechanisms are likely responsible for these effects.

## 6. Concluding Remarks

The current review reports recent experimental data supporting the role of honey as an immunomodulatory agent in cancer. These findings are derived from both in vitro and in vivo studies using different cancer types and preclinical models. Given the multiple bioactive compounds present in honey, it is not perhaps surprising that both pro-inflammatory as well as anti-inflammatory properties have been demonstrated for these constituents. The overall net changes induced by honey and its polyphenolic constituents alter the tumor microenvironment, reduce angiogenesis and re-program immune cells, making them more hostile to the continued growth and metastasis of cancer cells.

Despite these promising data, additional research is needed to further extend the current findings and identify the precise mechanisms by which honey induces its immunomodulatory effect. The recent demonstration of flavonoid compounds preferentially interacting with IL-6R, thus inhibiting its binding to IL-6 ligand and subsequent STAT3 activation [17], highlights a new mechanism by which these compounds can affect this critical signaling pathway. The utilization of appropriate preclinical cancer models to dissect the underlying mechanisms in fine detail is still required before potentially moving to clinical trials. Investigating the use of honey or its flavonoid constituents to boost the efficacy of cancer immunotherapy by checkpoint inhibitors would be quite rewarding. Moreover, combining the demonstrated efficacy of flavonoids with improved targeting to cancer tissue offers a possible way to enhance their anti-tumor action. A potentially exciting way that this can achieved is through the use of nanocarriers to improve the bioavailability and targeting efficacy of different flavonoid compounds [113]. Only then could we validate the application of honey and flavonoid compounds as preventative and therapeutic agents in cancer.
